# Obituary Brian P. Kavanagh, MD

**DOI:** 10.1186/s13054-019-2556-9

**Published:** 2019-08-08

**Authors:** John C. Marshall, Arthur S. Slutsky

**Affiliations:** 0000 0001 2157 2938 grid.17063.33 St. Michael’s Hospital, University of Toronto, Toronto, Canada

Critical care lost a scientific giant and a treasured colleague and friend when Brian Kavanagh passed away on June 15 at the age of 57. He was brilliant, complicated, and revered by patients and their families, as well as students and colleagues around the world.

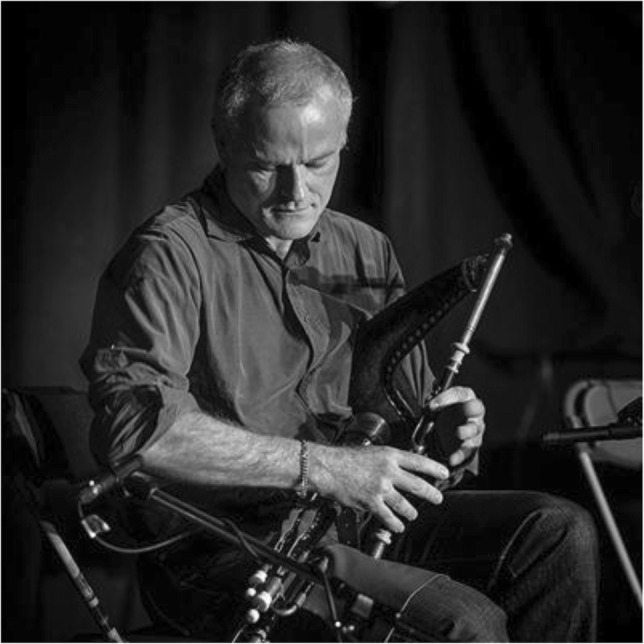



Brian was born in Dublin in 1962. His Irish upbringing imparted a passion for literature and music and honed in him a uniquely Irish sense of humor. Following a residency in internal medicine, he moved to Canada in 1989, undertaking a residency in anesthesia and a fellowship in critical care at Stanford. He began his critical care career at the Toronto General Hospital and moved to the Hospital for Sick Children in 1999. Over the ensuing two decades, he left an indelible mark.

He was the Chair of the Department of Anesthesia at the University of Toronto and of the Critical Care Canada Forum (CCCF), held in Toronto each autumn. He ran a productive laboratory that made important contributions to our understanding of the role of carbon dioxide during mechanical ventilation and developed new approaches to limit ventilator-induced lung injury. He mentored countless research trainees from around the world and was cherished as a clinician and an educator.

The skeletal details of a biography do not capture the essence of a complex human being and do not do justice to the impact he has had on the lives of others—patients and their families, Irish musicians, academic leaders, students, colleagues, family, and friends. This impact is better reflected through the outpouring of reminiscences, anecdotes, and condolences that followed his death.

A reminiscence from the UK: “A generous friend I never understood but will miss terribly.” That speaks to so many who worked with Brian in an academic sphere. He was unrelentingly smart and did not suffer platitudes or sloppy thinking, particularly from those in our field who occupied a position of influence or authority. He relished disagreement, arranging debates at the CCCF, for example, that pitted Martin Tobin against Gordon Guyatt debating evidence-based medicine or Charles Natanson against Emmanuel Rivers debating industry engagement in acute care research. The goal was not to draw blood, but rather to challenge the brightest and the smartest to think more deeply about the themes that had defined their academic careers, and to give the audience a peek into the assumptions and thinking processes underlying major issues.

Brian was caustic and was caustic in direct proportion to the place his targets held within the critical care hierarchy. A Toronto intensivist recalls an oral exam with a local trainee; asked a challenging question on the physiology of ARDS, the trainee spluttered and fell short. Brian’s response was as supportive of the trainee as it was dismissive of his teacher: “You answered that question on ARDS poorly, but Dr. X here probably couldn’t have answered it at all.” He was intolerant of sloppy, simplistic thinking and even more intolerant of those of us who deigned to put forward such ideas. As one colleague said, “It was only two days after a discussion with Brian that you realized that his comment to you was intended to be an insult.”

Brian changed our way of seeing the world. He challenged evidence-based medicine, showed us how carbon dioxide may be beneficial, helped describe new mechanisms of lung injury, and, most recently, focused our attention on the possibility that ventilation with negative abdominal pressure could mitigate ventilator-induced lung injury. He was a superb physiologist, but his brilliance extended far beyond physiology. With his incisive intellect, and his wry (and rye) sense of humor, he addressed provocative questions, co-authoring papers with titles like “Declaration of conflicts of interest: a ‘crooked’ line towards scientific integrity” and “Negative trials in critical care: why most research is probably wrong.” And he also tackled difficult ethical questions such as withholding and withdrawing treatment.

Brian mentored a new generation of critical care scientists whose words speak eloquently of his influence. “He opened doors for me and encouraged me to take on challenges I might have otherwise have passed up … We cared for Brian in the ICU … for the last 2 weeks of his life. That was a great privilege, but also heartbreaking. It was obvious then that Brian was going to die from this disease - but we thought that we would have a few more months with him. The end came quickly and somewhat unexpectedly when an infectious complication arose and he decided to decline invasive support for this. Even at the end, Brian was his best self … still working on manuscripts, planning future experiments and mentoring all around him. My favourite quote from the RN report on ICU rounds: ‘Dr. Kavanagh only slept a few hours last night - he was up too late chatting with the on-call ICU fellow and giving him scientific and career advice till the small hours of the morning.’”

Brian also mentored senior faculty as well. “In the mid-90’s the Chair of the Department of Anesthesiology asked me (AS) to mentor Brian when he returned from training at Stanford. It soon became clear how special and how talented Brian was. I think that I helped him a little bit at first, but I soon found myself turning to Brian for advice on a host of matters. He was strategic and was always thinking 3, 4, or 5 steps ahead. The mentor quickly became the mentee!”

From a junior faculty member: “Among the many lessons he taught me, the most important was the priority of understanding mechanisms of injury and treatment effect in individual patients. I remember most distinctly about Brian two things: he was present, and he aimed for perfection. When I started trying to find my way into physiology, Brian was an essential and constant guide and support—he opened up key opportunities and connected me to key people. He showed up for early morning thesis committees and joined me to celebrate graduation. He was also devoted to perfecting the art of scientific writing; I recall prolonged deliberations over the choice of wording in response to reviewers for an early breakthrough paper. He showed me how to wordsmith a grant application until each word represented a profoundly weighty choice.”

“Brian was an outstanding doctor, researcher, mentor and a role model for any of the lucky individuals who had the opportunity to meet him … but this is only a small part of what he was … the other amazing part is that he was a genuinely good and generous human being, always trying to share the extent of his possibilities (which were beyond imagination). He would listen and consider you the same if you are a first year medical student or an internationally recognized Professor. He had such a sharp sense of humor that it was challenging not to laugh out loud when he was making little comments … I hope he felt how much we loved him.”

“I was fortunate to learn from Brian as a Paediatric trainee, but even more fortunate that he was on service when my own son landed up in the PICU. I will be forever grateful for his kindness, humility and exceptional care.” Brian was more than an insightful and generous scientist: he was a dedicated and humble clinician. His abrasiveness as a thought leader was overshadowed by his ferocious and passion for those who entrusted him with their lives, and they voiced his impact at his memorial service. A particularly moving tribute came from a family whose child had died in the ICU. As it became clear that the child would not recover, the family asked that friends and loved ones send the saddest songs they knew to honor the life of their child. Brian sent an Irish lament, but more than this, followed it up 2 years later with a recorded version on the uilleann pipes, along with an upbeat ending—music and culture interwoven with a lasting memory and a profoundly moving gift to a grieving family.

That sense of the interplay of science and art exemplified Brian’s life. A gifted performer on the uilleann pipes, he found a home in a Toronto community of musicians who played Irish music and who drank Guinness and Irish whiskey at pubs around Toronto. The sad but profound sense of the music seemed to mirror a world view—deep, intense, and imperfect. Brian was a poet, and this same sensibility is reflected in the words of one of his favorite writers, W.B. Yeats, in his poem, “An Irish Airman Foresees his Death”:

Nor law, nor duty bade me fight,

Nor public man, nor cheering crowds,

A lonely impulse of delight

Drove to this tumult in the clouds;

I balanced all, brought all to mind,

The years to come seemed waste of breath,

A waste of breath the years behind

In balance with this life, this death.

Brian brought the same Irish sensibility of passion and acceptance that he brought to his science to the terminal stages of cancer that took his life. Less than 24 h before his passing, he was updating his curriculum vitae with his latest acknowledgment—a prestigious CIHR Tier one Canada Research Chair. As his daughter, Aifric, said at this memorial ceremony—“He taught me how to live; he also taught me how to die.”

Brian is survived by all who knew and were better for knowing him—his colleagues and friends in an international community of critical care; his dear friends in a community of Irish poets and musicians; the women who loved him, Hillary, Hannah, and Briseida; his daughters, Daire and Aifric; and a global community of those who care for the critically ill and have gained from knowing him.

*Go raibh Maith Agat Mo Chara –* thank you dear friend.

We are all better for the time you spent with us.

